# Draft genome sequence of a novel species of *Winogradskyella* (strain PC D3.3) isolated from a Red Antarctic macroalgae

**DOI:** 10.1128/mra.00055-26

**Published:** 2026-03-16

**Authors:** Jojy John, Amit Kumar, Jenifer Alvarez, Radhakrishnan Mannikam, Luis Vargas-Chacoff, Sergio Leiva Poveda

**Affiliations:** 1 Department of Biological Sciences, Clemson University 2545, Clemson, South Carolina, USA; 2 Center for Climate Change Studies, Sathyabama Institute of Science and Technology 119670, Chennai, Tamil Nadu, India; 3 Faculty of Sciences, Institute of Biochemistry and Microbiology, Universidad Austral de Chile 28040, Valdivia, Chile; 4 Center for Drug Discovery and Development, Sathyabama Institute of Science and Technology 119670, Chennai, Tamil Nadu, India; 5 Faculty of Sciences, Institute of Marine and Limnological Sciences, Universidad Austral de Chile 28040, Valdivia, Chile; 6 Instituto Milenio Biodiversidad de Ecosistemas Antárticos y Sub-Antárticos (BASE), Universidad Austral de Chile 28040, Valdivia, Chile; University of Southern California, Los Angeles, California, USA

**Keywords:** Antarctica, *Winogradskyella*, macroalgae, *Plocamium cartilagineum*, draft genome, Flavobacteriia

## Abstract

We report the draft genome sequence of *Winogradskyella* sp. nov. PC D3.3, a Flavobacteriia isolated from the surface of the red macroalgae *Plocamium cartilagineum* collected in King George Island, Antarctica. The genome assembly comprised 4,212,977 bp, with 33.32% G + C content and 3,748 coding sequences.

## ANNOUNCEMENT

During a survey of culturable bacteria from Antarctic macroalgae, a strain of *Winogradskyella*, designated PC D3.3, was isolated from the red alga *Plocamium cartilagineum*. Members of *Winogradskyella* (Flavobacteriaceae) are gram-negative, rod-shaped, and produce non-diffusible, yellow to orange pigments (1). They are widespread in marine environments (1) and are commonly associated with macroalgae (2–4), but reports from Antarctic macroalgae remain scarce (5, 6), underscoring the value of sequencing strain PC D3.3.

PC D3.3 was isolated from the surface of a healthy *P. cartilagineum* thallus collected in the subtidal zone of Shoa Island (62°12′12″S, 58°56′37″W), King George Island, Antarctica, on marine agar 2216 (BD) at 20°C. Microbiological processing of macroalgal samples followed Sánchez Hinojosa et al. (6). Colonies were purified by repeated streaking, and purity was verified by Gram staining and colony morphology. Genomic DNA was extracted from 72 h marine broth cultures using the GeneJET Genomic DNA Purification Kit and quantified with a Qubit fluorometer. Whole-genome sequencing was performed at AUSTRAL–omics (Valdivia, Chile). Libraries were prepared from 400 ng DNA with the ONT Native Barcoding Kit 24 V14 and sequenced on a MinION using a Flongle (R10.4.1) flow cell. Bead-based cleanups were performed according to the ONT protocol, without additional size selection. Reads were basecalled with Dorado v0.5.1 (high-accuracy model; https://github.com/nanoporetech/dorado), assessed for quality with NanoPlot v1.42.0 (7), and reads with *Q* < 7 were discarded. *De novo* assembly was performed with Flye v2.9.1 (8), and genome annotation was performed with Prokka v1.14.6 (9) and DRAM (10). Carbohydrate-active enzymes were identified in DRAM using dbCAN HMM profiles (*E*-value < 1 × 10⁻¹⁵; coverage > 0.35). Average nucleotide identity (ANI) and average amino acid identity were calculated with FastANI (11) and CompareM (https://github.com/donovan-h-parks/CompareM), and digital DNA-DNA hybridization values were estimated with the GGDC server (12). Default parameters were used unless otherwise specified.

The complete 16S rRNA gene was retrieved from the assembled genome and compared against EzBioCloud version 2025.04.21 (https://www.ezbiocloud.net/) (13). PC D3.3 showed 98.14% sequence similarity to *Winogradskyella ludwigii* HL116^T^ (accession no. JABFDF000000000) and 97.85% to *Winogradskyella sediminis* S5-23-3^T^ (accession no. KF312887). At the whole-genome level, PC D3.3 was closest to *Winogradskyella psychrotolerans* D3R19 (GCF_018860565.1; 85.8% ANI), followed by *Winogradskyella thalassocola* DSM 15363 (GCF_900099995.1; 85.4% ANI). Digital DNA-DNA hybridization (formula 2) yielded a maximum value of 29.0% with *W. psychrotolerans* D3R19, far below the 70% species threshold. These results suggest that PC D3.3 is likely a novel species within the genus *Winogradskyella*.

Oxford Nanopore sequencing produced 91,978 reads (719.4 Mb total) with a raw read N50 of 12,793 bp, and the genome of strain PC D3.3 was assembled into a single 4.21 Mb circular contig (assembly N50 = 4.21 Mb). The genome size is 4,212,977 bp, with 33.32% G + C content. The draft genome contains 3,748 coding sequences and 61 RNA genes (12 rRNA, 48 tRNA, and 1 transfer-messenger RNA). The genome encodes diverse polysaccharide-degrading enzymes for starch, hemicellulose (GH13 and GH43), alginate, and pectin (PL1, PL6, PL7, PL14, and PL17), suggesting that *Winogradskyella* sp. PC D3.3 contributes to the marine carbon cycle by utilizing polysaccharides from its algal host.

## Data Availability

All data are available under BioProject PRJNA1381634. Raw reads are deposited as accession SRS27497605, the genome assembly under GenBank accession JBSYWG000000000, and DRAM annotations at https://doi.org/10.6084/m9.figshare.31259503. This is the first version of the genome.

## References

[1] Nedashkovskaya OI, Kim SB. 2016. Winogradskyella, p 1–21. *In* Trujillo ME, Dedysh S, DeVos P, Hedlund B, Kämpfer P, FA Rainey, Whitman WB (ed), Bergey’s manual of systematics of archaea and bacteria. John Wiley & Sons.

[2] Kurilenko VV, Romanenko LA, Isaeva MP, Svetashev VI, Mikhailov VV. 2019. Winogradskyella algae sp. nov., a marine bacterium isolated from the brown alga. Antonie Van Leeuwenhoek 112:731–739. doi:10.1007/s10482-018-1207-5 30519785

[3] Nedashkovskaya OI, Kim SB, Han SK, Snauwaert C, Vancanneyt M, Swings J, Kim K-O, Lysenko AM, Rohde M, Frolova GM, Mikhailov VV, Bae KS. 2005. Winogradskyella thalassocola gen. nov., sp. nov., Winogradskyella epiphytica sp. nov. and Winogradskyella eximia sp. nov., marine bacteria of the family Flavobacteriaceae. Int J Syst Evol Microbiol 55:49–55. doi:10.1099/ijs.0.63307-0 15653852

[4] Nedashkovskaya OI, Kukhlevskiy AD, Zhukova NV. 2012. Winogradskyella ulvae sp. nov., an epiphyte of a Pacific seaweed, and emended descriptions of the genus Winogradskyella and Winogradskyella thalassocola, Winogradskyella echinorum, Winogradskyella exilis and Winogradskyella eximia. Int J Syst Evol Microbiol 62:1450–1456. doi:10.1099/ijs.0.032219-0 21841007

[5] Barreto CC, Duarte RTD, Lima DV, de Oliveira Filho EC, Absher TM, Pellizari VH. 2017. The cultivable microbiota associated with four antarctic macroalgae. OAJMB 2:000122. doi:10.23880/OAJMB-16000122

[6] Sánchez Hinojosa V, Asenjo J, Leiva S. 2018. Agarolytic culturable bacteria associated with three antarctic subtidal macroalgae. World J Microbiol Biotechnol 34:73. doi:10.1007/s11274-018-2456-1 29785671

[7] De Coster W, Rademakers R. 2023. NanoPack2: population-scale evaluation of long-read sequencing data. Bioinformatics 39:btad311. doi:10.1093/bioinformatics/btad311 37171891 PMC10196664

[8] Kolmogorov M, Yuan J, Lin Y, Pevzner PA. 2019. Assembly of long, error-prone reads using repeat graphs. Nat Biotechnol 37:540–546. doi:10.1038/s41587-019-0072-8 30936562

[9] Seemann T. 2014. Prokka: rapid prokaryotic genome annotation. Bioinformatics 30:2068–2069. doi:10.1093/bioinformatics/btu153 24642063

[10] Shaffer M, Borton MA, McGivern BB, Zayed AA, La Rosa SL, Solden LM, Liu P, Narrowe AB, Rodríguez-Ramos J, Bolduc B, Gazitúa MC, Daly RA, Smith GJ, Vik DR, Pope PB, Sullivan MB, Roux S, Wrighton KC. 2020. DRAM for distilling microbial metabolism to automate the curation of microbiome function. Nucleic Acids Res 48:8883–8900. doi:10.1093/nar/gkaa621 32766782 PMC7498326

[11] Jain C, Rodriguez-R LM, Phillippy AM, Konstantinidis KT, Aluru S. 2018. High throughput ANI analysis of 90K prokaryotic genomes reveals clear species boundaries. Nat Commun 9:5114. doi:10.1038/s41467-018-07641-9 30504855 PMC6269478

[12] Meier-Kolthoff JP, Auch AF, Klenk H-P, Göker M. 2013. Genome sequence-based species delimitation with confidence intervals and improved distance functions. BMC Bioinformatics 14:60. doi:10.1186/1471-2105-14-60 23432962 PMC3665452

[13] Chalita M, Kim YO, Park S, Oh H-S, Cho JH, Moon J, Baek N, Moon C, Lee K, Yang J, Nam GG, Jung Y, Na S-I, Bailey MJ, Chun J. 2024. EzBioCloud: a genome-driven database and platform for microbiome identification and discovery. Int J Syst Evol Microbiol 74:006421. doi:10.1099/ijsem.0.006421 38888585 PMC11261700

